# Sentinel node biopsy should be supplemented by axillary sampling in patients with small breast cancers

**DOI:** 10.1186/1477-7800-2-27

**Published:** 2005-11-28

**Authors:** A Adwani, SR Ebbs, S Burton, S Lowe

**Affiliations:** 1 Breast Surgery Department, Mayday University Hospital, London Road, Croydon, Surrey, CR7 7YE, UK; 2 Radiology Department, Mayday University Hospital, London Road, Croydon, Surrey, CR7 7YE, UK

**Keywords:** Early breast cancer, sentinel node biopsy, axillary sampling, guided axillary sampling

## Abstract

Axillary clearance provides important prognostic information but is associated with significant morbidity. Sentinel node biopsy can provide staging .141 patients with node negative early breast cancers-tumour size less than 1.5 cm measured clinically or by imaging had guided axillary sampling (sentinel lymph node biopsy in combination with axillary sampling). Four node axillary sampling improved the detection rate of axillary node metastases by 13.6% as compared to blue dye sentinel node biopsy alone. Positive sampled nodes strongly indicated the likelihood of further metastatic being revealed by axillary dissection (67%). Negative sampled nodes in combination with a positive sentinel node biopsy were associated with a much lower rate of further nodal involvement in the axillary clearance (8%).

## Introduction

For patients with early breast cancer axillary lymph node status is the single most important prognostic indicator enabling decisions regarding adjuvant systemic treatment [1,2]. Axillary clearance remains the most accurate method of staging the axilla but the procedure is associated with significant morbidity [3-5].

The likelihood of lymph node metastases is a function of tumour size, particularly for small cancers, the incidence of which is increasing [6,7]. Elective axillary dissection may be over-treatment for a high proportion of such cases. Many studies have indicated that the sentinel node biopsy (SNB) concept is applicable to patients with early breast cancer [8] and Veronesi has suggested that the procedure might be limited to tumours of 15 mm or less [9]. SNB is being validated in randomised trials NSABP B32, ACOS-OG and the ALMANAC study but it will be some years before long-term results are available. These studies, however, include larger tumours and will require subset analysis to study the outcome for small tumours.

The SNB concept suggests removal of the single first node draining the tumour lymph however many of the reports have described excision of multiple lymph nodes [10-15].

Previous reports of 4 or 5 node axillary sampling have suggested accuracy rates equivalent to SNB [13-15]. We have sought to determine whether the planned removal of at least 4 axillary nodes increases the accuracy of axillary staging above that of the removal of the sentinel node.

## Materials and methods

Between January 1998 and December 2002, patients with clinically node negative early breast cancers-tumour size less than or equal to 15 mm (measured clinically or by imaging) had guided axillary sampling (sentinel lymph node biopsy followed by excision of further nodes to ensure a minimum of four nodes had been removed).

### Sentinel node biopsy technique

Sentinel node biopsy (SNB) was performed using the dye technique only without the adjunct of radioisotope labelling and gamma probe. Five mls of patent blue dye was infiltrated peritumorally or subcutaneously in palpable cancers, around the guide wire tip in impalpable cancers or into the cavity wall when performed as a secondary procedure to initial tumour excision.

After 5 minutes, a small low axillary incision was used to identify blue stained afferent lymphatics which were traced to the first blue stained sentinel node or nodes. All blue stained nodes were excised and sent in a separate pot labelled sentinel nodes.

### Axillary sampling technique

Following excision of the sentinel node(s), at least three further palpable lymph nodes were excised, labelled as axillary node sample (ANS) and sent separately for histological analysis.

Wide local excision of the breast tumour was performed in the usual way, only after sentinel node biopsy and axillary node sampling were completed. All lymph nodes were examined histopathologically using H&E staining. A level three completion axillary clearance (ANC) was subsequently performed for patients who had metastatic tumour cells identified histologically in the sentinel node or sampled axillary lymph nodes. Further treatment included radiotherapy and adjuvant hormonal or chemotherapy as per local guidelines.

## Results

142 operations were performed on 141 patients whose ages ranged from 30 to 85 years of age (mean = 60). 83 patients with 84 cancers were referred from the National Health Service Breast Screening Programme (NHSBSP). Of these 5 lesions were palpable. Of the remaining 58 patients not from screening, 18 had impalpable cancers. The 45 palpable cancers ranged in size from 5 to 15 mm (mean 11 mm). Of 98 lesions measurable on mammography, size ranged from 4 to 15 mm (mean 10 mm). The 108 cancers measured on ultrasound ranged from 2.6 to 15 mm (mean 8.5 mm).

Ninety-nine lesions required localisation to guide their breast excision. The localisation was performed with mammographic guidance in 33 cases and with ultrasound guidance in 66. In 27 cases, axillary surgery was performed at a separate operation to the breast resection.

**Figure 1:**
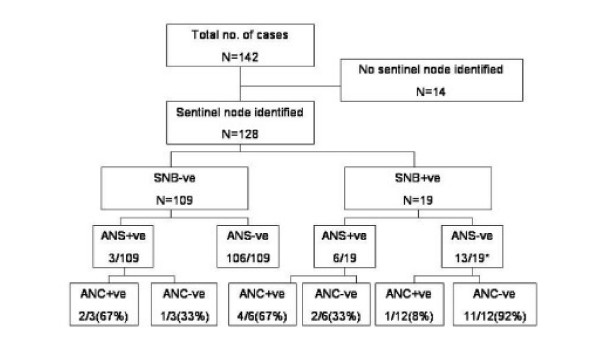
**Incidence of nodal disease for patients with small breast cancers**. SNB – sentinel node biopsy ANS – axillary node sample ANC – axillary node clearance * One patient with positive SNB but negative ANS received reduced dose radiotherapy to the axilla rather than undergoing ANC.

One hundred and twenty eight cases of the 142 (90.14%) had successful identification of a sentinel node (Figure 1). The median sentinel node biopsy count was 1(mean = 1.47), median axillary node count was 4 (mean = 4.65) and the median total node count for SNB and ANS was 6 (mean = 6.13).

The finding of a negative sentinel node was correctly predictive of no further nodal metastasis in 106 of 109 cases (97%). In the three cases with a negative sentinel node but positive sampled nodes, the axillary clearance found further positive nodes in 2 of 3 cases. When the sentinel node was positive (19 of 128 cases, 14.8%), the axillary node sampling was also positive in 32% (6 of 19 cases).

The finding of negative nodes in the axillary sampling after a positive sentinel node indicated that axillary clearance was unlikely to reveal further positive nodes (1 of 12 cases; 8%). The combination, however, of a positive sentinel node and positive axillary sampling, indicated that axillary clearance was highly likely to be positive (4 of 6 cases; 67%).

In 14 patients a sentinel node could not be identified (9.9%). In these patients, 68 nodes were removed (3–5 nodes per patient). In two patients, a total of only 3 nodes were found on histological examination. All other patients had 4 or more nodes excised. Only one of these 13 patients had axillary metastasis identified and this was in a single lymph node – subsequent axillary clearance failed to reveal any further malignant nodes in this patient.

Twenty five of one hundred and forty two tumours (18%) tumours were found to be greater than 15 mm in diameter on final histology (Table 1). Of these, 13 cases had positive nodes identified (13/25, 52%) with 11 of them having a positive sentinel node (11/13, 85%). Eight of 117 tumours with histological size less than or equal to 15 mm were found to be SNB positive with subsequent axillary sampling identifying 2 ANS positive cases (2/8, 25%) (Table 1).

**Table 1:** Axillary node status according to size and grade of tumour and presence of lymphovascular invasion

**Grade**	**LVI**	**Number of positive nodes/ total nodes (%)**			
		
		**Tumour < 11 mm**	**Tumour 15 – 11 mm**	**Tumour >15 mm**	**All tumours**
**1**	**LVI+**	0/3 (0%)	1/1 (100%)	0	1/4 (25%)
n = 55	**LVI-**	0/30 (0%)	2/15 (13%)	2/6 (33%)	4/51 (8%)
**2**	**LVI+**	1/2 (50%)	1/3 (33%)	4/4 (100%)	6/9 (67%)
n = 67	**LVI-**	1/22 (5%)	1/27 (4%)	5/9 (56%)	7/58 (12%)
**3**	**LVI+**	0	0/2 (0%)	1/2 (50%)	1/4 (25%)
n = 20	**LVI-**	2/7 (29%)	0/5 (0%)	1/4 (25%)	3/16 (19%)

G = grade (1–3)

LVI = Lymphovascular invasion (positive (+) or negative (-)

Sixty-eight tumours measured less than 11 mm. Of these, 64 were SNB negative and 4 were SNB positive (3 ANS negative, 1 ANS positive) (Table 1).

Of 141 tumours where the ER status was known, 13 were ER negative and of these 3 (23%) were node positive at surgery, whilst 128 ER positive and of these15 (12%) node positive at operation. The single ER unknown patient was node positive (Table 1).

## Discussion

Axillary node dissection has previously been regarded as an essential component of the management of patients with early breast cancer [16]. Whilst neither reaches the accuracy of axillary clearance, the low error rate of sentinel node biopsy and unguided axillary sampling has lead to proponents arguing the appropriateness of both techniques [10,12,13]. If it is accepted that the necessity of full axillary clearance is now an outmoded concept inappropriate for many patients with small breast cancers, then several issues still require clarification to ensure the maximum efficiency of limited axillary surgery. Whilst there are limitations of our study, we have sought to explore some of these aspects.

In this study, using blue dye was successful in revealing a sentinel node in 90% of cases. Finding a negative sentinel node was highly predictive of further nodes in the axilla being clear of cancer. However the simple extension of removing four nodes in total ensured that the 3% of cases with positive nodes but a negative SNB were correctly identified. We would therefore recommend that SNB techniques should remove a minimum of 4 nodes.

For patients with a positive sentinel node, the finding of additional positive nodes in the axillary sample strongly indicated the likelihood of further positive nodes in the axilla, therefore, justifying an axillary clearance. If the SNB was positive and the ANS negative, the likelihood of further malignant nodes being revealed by an axillary clearance was much lower (1/12, 8%) but with only this limited evidence we still feel that this figure justifies proceeding to ANC.

Many parameters are correlated with the likelihood of axillary node involvement [7,17,18]. We have examined subgroups of our patients to try and determine whether there is a group of patients whose pre-operative parameters mean that even limited axillary surgery might be safely avoided. Our previously reported experience of palpable tumours has shown how difficult it is to predict the histological size of invasive cancers [19]. This study has demonstrated that even when clinical, ultrasound and mammographic measurements indicate a tumour size of 15 mm or less, this was incorrect in 25 of 142 (18%) of patients. It has been reported that pre-operative grade is not reliably assessed without numerous core biopsies which may be difficult on small cancers [20-22]. Lymphovascular invasion can only be evaluated on resected tumours [23]. Another pre-operative parameter that might be accurately evaluated is oestrogen receptor status on core biopsies [24-26]. However, our results demonstrate that this alone was not useful.

An argument might be made to perform staged surgery and look at the possible parameters on the formal histology of the resected tumour. Tables 1 shows that in this study, none of the parameters was able to reliably indicate a group of patients with a low level (under 5%) of axillary node involvement with the exception of grade 1 tumours less than or equal to 15 mm or grade 2 tumours under 11 mm, both without lymphovascular invasion.

We also considered whether there could be a subgroup of patients with small tumours whose pre-operative parameters mean limited axillary surgery is inappropriate and patients should proceed directly to radical axillary surgery. However, revisiting Table 1 indicates that even patients with the most unfavourable histology have at least a 50% chance of being node negative supporting the concept of initial limited axillary surgery.

In this study, additional axillary node sampling identified 13.6% (3/22) more node positive patients. Similarly, the identification of positive sampled nodes in addition to a positive sentinel node strongly predicted for further axillary node involvement.

In summary, we propose the simple expedient of sampling four nodes to increase the accuracy of sentinel node biopsy.

## References

[B1] (1998). Tamoxifen for early breast cancer: an overview of the randomised trials. Early Breast Cancer Trialists' Collaborative Group. Lancet.

[B2] (1998). Polychemotherapy for early breast cancer: an overview of the randomised trials. Early Breast Cancer Trialists' Collaborative Group. Lancet.

[B3] Ivens D, Hoe AL, Podd TJ, Hamilton CR, Taylor I, Royle GT (1992). Assessment of morbidity from complete axillary dissection. Br J Cancer.

[B4] Kissin MW, Querci della Rovere G, Easton D, Westbury G (1986). Risk of lymphoedema following the treatment of breast cancer. Br J Surg.

[B5] Moffat FLJ, Senofsky GM, Davis K, Clark KC, Robinson DS, Ketcham AS (1992). Axillary node dissection for early breast cancer: some is good, but all is better. J Surg Oncol.

[B6] Cady B, Stone MD, Schuler JG, Thakur R, Wanner MA, Lavin PT (1996). The new era in breast cancer. Invasion, size, and nodal involvement dramatically decreasing as a result of mammographic screening. Arch Surg.

[B7] Carter CL, Allen C, Henson DE (1989). Relation of tumor size, lymph node status, and survival in 24,740 breast cancer cases. Cancer.

[B8] Hsueh EC, Hansen N, Giuliano AE (2000). Intraoperative lymphatic mapping and sentinel lymph node dissection in breast cancer. CA Cancer J Clin.

[B9] Veronesi U, Zurrida S, Galimberti V (1998). Consequences of sentinel node in clinical decision making in breast cancer and prospects for future studies. Eur J Surg Oncol.

[B10] Ahlgren J, Holmberg L, Bergh J, Liljegren G (2002). Five-node biopsy of the axilla: an alternative to axillary dissection of levels I-II in operable breast cancer. Eur J Surg Oncol.

[B11] Barthelmes L, Al-Awa A, Murali-Krishnan VP, Crawford DJ (2002). The role of lymph node sampling and radiotherapy in the management of the axilla in early breast cancer. Breast.

[B12] Chetty U, Jack W, Prescott RJ, Tyler C, Rodger A (2000). Management of the axilla in operable breast cancer treated by breast conservation: a randomized clinical trial. Edinburgh Breast Unit. Br J Surg.

[B13] Forrest AP, Everington D, McDonald CC, Steele RJ, Chetty U, Stewart HJ (1995). The Edinburgh randomized trial of axillary sampling or clearance after mastectomy. Br J Surg.

[B14] Hoar FJ, Stonelake PS (2003). A prospective study of the value of axillary node sampling in addition to sentinel lymph node biopsy in patients with breast cancer. Eur J Surg Oncol.

[B15] Macmillan RD, Barbera D, Hadjiminas DJ, Rampaul RS, Lee AH, Pinder SE, Ellis IO, Blamey RW, Geraghty JG (2001). Sentinel node biopsy for breast cancer may have little to offer four-node-samplers. results of a prospective comparison study. Eur J Cancer.

[B16] Fentiman IS, Mansel RE (1991). The axilla: not a no-go zone. Lancet.

[B17] Barth A, Craig PH, Silverstein MJ (1997). Predictors of axillary lymph node metastases in patients with T1 breast carcinoma. Cancer.

[B18] Jackson JS, Olivotto IA, Wai MDE, Grau C, Mates D, Ragaz J (2000). A decision analysis of the effect of avoiding axillary lymph node dissection in low risk women with invasive breast carcinoma. Cancer.

[B19] Pain JA, Ebbs SR, Hern RP, Lowe S, Bradbeer JW (1992). Assessment of breast cancer size: a comparison of methods. Eur J Surg Oncol.

[B20] Di Loreto C, Puglisi F, Rimondi G, Zuiani C, Anania G, Della Mea V, Beltrami CA (1996). Large core biopsy for diagnostic and prognostic evaluation of invasive breast carcinomas. Eur J Cancer.

[B21] Connor CS, Tawfik OW, Joyce AJ, Davis MK, Mayo MS, Jewell WR (2002). A comparison of prognostic tumor markers obtained on image-guided breast biopsies and final surgical specimens. Am J Surg.

[B22] McIntosh SA, Panchalingam L, Payne S, Miller ID, Sarkar TK, Hutcheon AW, Heys SD (2002). Freehand core biopsy in breast cancer: an accurate predictor of tumour grade following neoadjuvant chemotherapy?. Breast.

[B23] Denley H, Pinder SE, Elston CW, Lee AH, Ellis IO (2001). Preoperative assessment of prognostic factors in breast cancer. J Clin Pathol.

[B24] Harris K, Morafa I, Thomas V, Mokbel K (2004). Core biopsy is accurate in determining the hormone receptor status of early breast cancer. Am J Surg.

[B25] Douglas-Jones AG, Collett N, Morgan JM, Jasani B (2001). Comparison of core oestrogen receptor (ER) assay with excised tumour: intratumoral distribution of ER in breast carcinoma. J Clin Pathol.

[B26] Shannon J, Douglas-Jones AG, Dallimore NS (2001). Conversion to core biopsy in preoperative diagnosis of breast lesions: is it justified by results?. J Clin Pathol.

